# Case Report: Long-Acting Oral Cariprazine

**DOI:** 10.3389/fpsyt.2022.876003

**Published:** 2022-04-27

**Authors:** Emanuela Dyrmishi, Marco De Pieri, Marco Ferrari, Rafael Traber, Matteo Preve, Luca De Peri, Emilio Bolla

**Affiliations:** ^1^Organizzazione Sociopsichiatrica Cantonale, Mendrisio, Switzerland; ^2^PhD Program in Clinical and Experimental Medicine and Medical Humanities, University of Insubria, Varese, Italy; ^3^Center for Research in Medical Pharmacology, Varese, Italy

**Keywords:** long acting antipsychotics, cariprazine, therapeutic alliance, personalized medicine, adherence–compliance–persistence

## Abstract

**Introduction:**

Cariprazine is a third-generation antipsychotic, approved for the treatment of schizophrenia and bipolar disorder and used off-label for schizoaffective disorder and treatment-resistant depression. Cariprazine is a partial agonist at dopamine receptors D2 and D3 and serotonin receptor 5HT1A and an antagonist at serotonin receptors 5HT2B and 5HT2A. It is metabolized by CYP3A4 in desmetyl-cariprazine and didesmethyl-cariprazine, both active metabolites with a half-life of 1–2 days and 2–3 weeks, respectively.

**Case Report:**

Here we show the cases of 3 outpatients diagnosed with bipolar I disorder (two patients) and schizoaffective disorder (one patients) and characterized by low adherence to treatment, satisfactory cognitive and personal functioning and average disease severity to whom we administered cariprazine as a monotherapy, on a two-times a week schedule (i.e., every 72–96 h). We evaluated response to treatment and disease remission according to conventional definitions, using rating scales BPRS, PANSS and BDI-II. Two-times a week treatment was set either after a disease relapse (one patient), after a sustained remission obtained with daily administration of cariprazine (one patient) or since our first evaluation (one patient). After 4 weeks of treatment all three patients satisfied criteria for response to treatment and remission, a result that was sustained for 8 (in one patients) and 12 months (in other two patients) and still ongoing.

**Discussion:**

Reported results support our hypothesis that long half-lives of cariprazine and its metabolites provide an adequate therapeutic response with a two-times a week administration. In selected patients, cariprazine administered as a “oral long-acting” seems effective in treating acute episodes of illness and in sustaining remission, combining advantages of oral and long-acting injectable antipsychotics concerning therapeutic alliance.

## Introduction

Cariprazine (CAR) is a third-generation antipsychotic, approved for the treatment of schizophrenia (1, 2), acute-mixed mania (3, 4) and bipolar depression (5, 6), and used off-label for schizoaffective disorder and treatment resistant depression (7, 8). Cariprazine is effective on the cognitive and negative psychopathological dimensions, more than on positive symptoms of psychosis (9, 10); furthermore it has a favorable profile regarding metabolic and extrapyramidal side effects (11, 12).

Cariprazine is a partial agonist at dopamine receptors D2 and D3 and at serotonin receptor 5HT1A and an antagonist at serotonin receptors 5HT2B and 5HT2A (13, 14). It is extensively metabolized by CYP3A4 (and to a lesser extent by CYP2D6), in desmetyl-cariprazine (DCAR) and didesmethyl-cariprazine (DDCAR). The half-lives of cariprazine, desmetyl-cariprazine and didesmethyl-cariprazine in humans are 1–3 days, 1–2 days, and 2–3 weeks, respectively (15, 16). Both metabolites are active on D3, D2, 5-HT1A, 5-HT2A receptors (13, 17).

Treatment with long acting antipsychotics, in the injectable formulation (LAI), provides many benefits compared to oral antipsychotics (OAP): it determines a reduction in relapse of the disease, drug discontinuation and hospitalization rates, a longer time to relapse, fewer hospital days per admission and a better adherence to treatment (18–20).

Unfortunately, LAI are often scarcely accepted by patients, making their prescription detrimental for therapeutic alliance, and only few second and third generation antipsychotics are available in this formulation.

In the case of LAI's the long acting properties are obtained through an extended-release formulation; there are instead two antecedents of psychotropics, administered through the oral route, whose long acting effect is based on the long half-life of the active principle itself.

Penfluridol, characterized by a half-life of 66 h, was used in the 70s and 80s on a weekly schedule for the treatment of schizophrenia (21, 22).

Fluoxetine has a half-life of 1–4 days and proved to be effective in the maintenance treatment of major depression and anxiety disorders when administered once a week. Several studies evidenced no significant differences in relapse rate, adherence and efficacy, when compared to daily administration (23, 24).

Here we report about the use of oral cariprazine 6 mg, administered on a two-times a week schedule, in selected patients characterized by scarce adherence to treatment, satisfactory cognitive and personal functioning and average disease severity.

In one case cariprazine was administered two-times a week since the beginning of the treatment, whereas in the other two cases after a period of daily administration.

In all three cases cariprazine was a monotherapy, patients indeed received no other antipsychotics or mood stabilizers, but benzodiazepines were allowed.

Clinical progression was evaluated with rating scales BPRS (brief psychiatric rating scale) (25), PANSS (positive and negative symptoms of schizophrenia) (26) and BDI-II (Beck depression inventory II) (27).

We defined response to treatment as a 50% reduction of the rating scale's score (28, 29). According to the work of Andreasen et al. on antipsychotic treatment, disease remission was defined as a score ≤ 3 for each item of BPRS and/or PANSS (30, 31); to this end we considered also a score ≤ 10 for BDI-II (27). Rating at each observation was given by the same evaluator, in order to avoid inconsistencies deriving from inter-rater variability.

Rationale for cariprazine use as a long-acting oral relies on the following points:

1. half-lives of cariprazine and its active metabolites are very long;
2. a dissociation occurs in pharmacokinetic attenuation between central dopamine D2 receptor occupancy and peripheral blood concentration of antipsychotics, meaning that drug effects at the level of CNS last longer than what could be expected according to the plasma level decline (32);
3. single-daily regimen administration of antipsychotics proved to be equivalent to multiple-daily regimen in terms of efficacy and safety, indicating that prolonging the administration interval of antipsychotics could not affect their clinical effects (33).

### Case 1

A 54 years old man, with a 30 years history of bipolar I disorder, characterized by both episodes of mania and depression, that was never appropriately treated due to scarce adherence. He received many treatments as an outpatient, and was prescribed valproate (maximum dosage 500 mg), fluoxetine (maximum dosage 40 mg), sertraline (maximum dosage 25 mg) and several benzodiazepines in the years. Antidepressants induced 2 times a switch to mania. He is graduated in economics science, and worked as an entrepreneur, his activities had mixed fortune, probably due to mood cycles.

He asked himself for an ambulatory evaluation due to depressed mood for 3 months, accompanied by insomnia, anxiety and irritability. He interrupted valproate some weeks before, due to concerns about weight gain and sedation. At the first visit mental status was characterized by irritable and depressed mood, abulia, anhedonia, insomnia, psychomotor agitation. Reality testing was partially compromised due to overvalued nihilistic and paranoid ideas, and complex attention was impaired.

According to DSM 5 criteria, a diagnosis of bipolar I disorder was confirmed, and current psychopathology was interpreted as a depressive episode with mixed features. A psychiatric admission was not needed, and therapeutic options were overtly discussed with the patient, including LAI administration.

Cariprazine was prescribed, in monotherapy, for a daily assumption for 3 months, 1.5 mg for the first week and then 3 mg, associated with flurazepam for insomnia in the first 4 weeks. After 1 week the patient developed akathisia, not requiring a specific treatment, self-remitting in 1 week.

The course was favorable, severity of disease gradually declined, in the 4 weeks control visit we witnessed both response to treatment and disease remission (see Table 1). After 4 more weeks he had a relapse, consisting in a new episode characterized by symptoms similar to the ones described above, but of lesser severity. We discovered that it was due to a complete withdrawal of cariprazine for the last month, due to stigma surrounding antipsychotics. After a successful psychoeducation on these drugs, we agreed on the administration of cariprazine 6 mg two-times a week, under supervision of a nurse, on Monday and Thursday morning (i.e., every 72 or 96 h). Outcome was positive, after 4 weeks criteria for response and remission were again met, and severity of the mild residual symptoms were similar to the one observed after daily treatment (see Table 1). In the course we observed a further improvement, remission was sustained after 8 months and still ongoing.

**Table 1:** Response to CAR treatment.

**Weeks of treatment**	**Rating scales score**	**Administrations schedule**					
		**Case 1**		**Case 2**		**Case 3**	
		**Daily**	**Two times a week**	**Daily**	**Two times a week**	**Daily**	**Two times a week**
Baseline	BPRS	58	53	80	24	–	52
2		51	48	64	23	–	42
4		40	43	48	21	–	35
8		36	37	32	21	–	27
16		53	35	27	20	–	27
32		–	33	–	22	–	25
52		–	–	–	19	–	22
Baseline	PANSS	–	–	142	35	–	–
2		–	–	115	35	–	–
4		–	–	87	33	–	–
8		–	–	58	32	–	–
16		–	–	48	31	–	–
32		–	–	–	32	–	–
52		–	–	–	31	–	–
Baseline	BDI-II	24	24	40	5	–	27
2		18	16	30	4	–	23
4		11	11	25	4	–	17
8		9	7	11	3	–	13
16		24	5	8	3	–	10
32		–	4	–	4	–	8
52		–	–	–	3	–	7

*Rating scales scorings (BPRS, brief psychiatric rating scale; PANSS, positive and negative symptoms of schizophrenia; BDI-II Beck depression inventory, 2nd version)*.

### Case 2

A 31 years old man, refugee in Switzerland, graduated in English literature, divorced, had no sons. He had a 10 years psychiatric history, characterized by 2 admissions in a psychiatric hospital, due to attempts to suicide by venous section. It was not possible to define his pharmacological history and at the moment of our evaluation he was not under treatment. He was sent to our ambulatory upon request of the general practitioner for behavioral abnormalities and alcohol abuse. At the first visit he looked bizarre, suspicious, puzzled, interpretative and displayed psychotic and depressive symptoms consisting of disorganized thought process, delusions of reference, anxiety, depressed mood and emotional lability. Clinical status satisfied both criteria A for schizophrenia and the criteria for a major depressive episode, according to DSM 5.

Considering the history of depression, the past occurrence of delusions in the absence of mood symptoms and the mental status at the first evaluation a diagnosis of schizoaffective disorder, depressive type, was formulated, based on DSM 5.

The patient was prescribed with daily cariprazine at a dosage of 1.5 mg for 1 week, then 3 mg for 2 weeks and eventually 4.5 mg. The course was favorable, delusional ideas vanished, disorganization of thoughts and behavior resolved as well as the depressive symptoms, and he met criteria for response and remission after 4 weeks. He further improved in the following period, and after 16 weeks since the beginning of the treatment he asked for its interruption. At this point we agreed on a two-times a week cariprazine 6 mg administration under nurse supervision (on Monday and Tuesday), that guaranteed a sustained remission for 1 year, that is still ongoing.

### Case 3

A 44 years old woman, with a 20 years psychiatric history as outpatient and inpatients, in some case subjected to mandatory admission. On the basis of DSM 5 criteria, she was diagnosed with a bipolar I disorder, with depressive and manic episodes characterized also by psychotic symptoms. He had multiple pharmacological attempts with full dose carbamazepine (up to 800 mg), lithium (often found to be not in therapeutic range), quetiapine (up to 600 mg), olanzapine (up to 20 mg), sertraline (50 mg), trazodone (up to 150 mg), all failed, mainly due to poor adherence. At our first evaluation, her mental status was characterized by conceptual disorganization, racing of thoughts, distractibility, psychomotor agitation, but also by loss of interest and abulia. Thought content included both grandiose and depressive ideas, such as feelings of sadness, worthlessness and hopelessness. Before this evaluation she have been prescribed with clozapine 25 mg 2 times a day, mirtazapine 45 mg in the evening and alprazolam 2 mg 3 times a day; she had spontaneously withdrawn clozapine for 3 weeks, was taking mirtazapine irregularly and alprazolam as scheduled. Therapeutic option was then discussed with the patients, stressing the need for an atypical antipsychotic, possibly in a LAI formulation; in the end we found an agreement on the assumption of cariprazine 6 mg two-times a week (Monday and Thursday) under nurse supervision. Outcome was favorable, response and remission were reached in 4 weeks, and persisted at the 1-year follow-up, that is still ongoing. Alprazolam was gradually discontinuated during the first 6 weeks of treatment.

## Discussion

We administered oral cariprazine 6 mg on a two-times a week schedule as a monotherapy to 3 patients diagnosed with schizoaffective (one patient) and bipolar disorder (two patients), all three characterized by low adherence to treatment, good cognitive, personal and interpersonal functioning and average disease severity (as indicated by baseline scores of rating scales BPRS, PANSS, and BDI-II). Moreover, they never had a consistent attempt at an antipsychotic treatment in the past, therefore the first-line was a 2nd or 3rd generation antipsychotic. Our working hypothesis was that, due the long half-lives of cariprazine, desmetyl-cariprazine, and didesmetyl-cariprazine, drug administration every 72–96 h provides an adequate therapeutic response.

In one case two-times a week treatment was set after a relapse due to lack of adherence, in one case after a sustained remission obtained with a daily administration of cariprazine and in one case since the beginning. At 4 weeks all three patients satisfied criteria for response to treatment and remission, a result that was sustained and is still ongoing. We detected no side effects, except for mild akathisia in one patient, self-remitting in about 1 week.

According to our knowledge this report represents the first case of administration of cariprazine as an oral long acting oral antipsychotic (LAO), and suggests the validity of this way of prescribing for psychotropics characterized by long half-lives, in line with similar studies on Penfluridol and Fluoxetine.

## Conclusion

In our opinion long-acting oral cariprazine could be a good option for outpatients characterized by low-average disease severity, high cognitive and interpersonal functioning, having problem of adherence to treatment and requiring a 2nd or 3rd generation antipsychotic. According to the different scenario represented by each case under study, long-acting oral cariprazine administration could be set since the beginning of a treatment, as a maintenance therapy or to cure an acute relapse.

Further controlled studies are needed to systematically explore this option, and particularly to assess the relapse rate, compared to a cohort receiving the drug according to the in-label schedule.

## Data Availability Statement

The raw data supporting the conclusions of this article will be made available by the authors, without undue reservation.

## Ethics Statement

Ethical review and approval was not required for the study on human participants in accordance with the local legislation and institutional requirements. The patients/participants provided their written informed consent to participate in this study. Written informed consent was obtained from the individual(s) for the publication of any potentially identifiable images or data included in this article.

## Author Contributions

MD conceptualized the manuscript. ED collected the clinical data. MD and ED drafted manuscript first version. RT, MP, MF, and EB participated in critical analysis and review of the manuscript. All authors read and approved the final manuscript.

## Conflict of Interest

The authors declare that the research was conducted in the absence of any commercial or financial relationships that could be construed as a potential conflict of interest.

## Publisher's Note

All claims expressed in this article are solely those of the authors and do not necessarily represent those of their affiliated organizations, or those of the publisher, the editors and the reviewers. Any product that may be evaluated in this article, or claim that may be made by its manufacturer, is not guaranteed or endorsed by the publisher.
